# Genetic diversity and ecology of coronaviruses hosted by cave-dwelling bats in Gabon

**DOI:** 10.1038/s41598-020-64159-1

**Published:** 2020-04-30

**Authors:** Gael Darren Maganga, Anaïs Pinto, Illich Manfred Mombo, Mankomra Madjitobaye, Antoine Mitte Mbeang Beyeme, Larson Boundenga, Meriadeg Ar Gouilh, Nadine N’Dilimabaka, Jan Felix Drexler, Christian Drosten, Eric Maurice Leroy

**Affiliations:** 1 Centre International de Recherches Médicales de Franceville (CIRMF), BP 769 Franceville, Gabon; 2 Université des Sciences et Techniques de Masuku (USTM), Institut National Supérieur d’Agronomie et de Biotechnologies (INSAB), BP 913 Franceville, Gabon; 3 Normandie Université, EA2656, Groupe de Recherche sur l’Adaptation Microbienne, 14000 Caen, France; 4 Charité-Universitätsmedizin Berlin, corporate member of Freie Universität Berlin, Humboldt-Universität zu, Berlin, Germany; 5 German Centre for Infection Research (DZIF), Heidelberg, Germany; 6 UMR (IRD 224/CNRS 5290/UM1-UM2), Institut de Recherche pour le Développement, Montpellier, France

**Keywords:** Ecology, Microbiology

## Abstract

Little research on coronaviruses has been conducted on wild animals in Africa. Here, we screened a wide range of wild animals collected in six provinces and five caves of Gabon between 2009 and 2015. We collected a total of 1867 animal samples (cave-dwelling bats, rodents, non-human primates and other wild animals). We explored the diversity of CoVs and determined the factors driving the infection of CoVs in wild animals. Based on a nested reverse transcription-polymerase chain reaction, only bats, belonging to the *Hipposideros gigas* (4/156), *Hipposideros* cf. *ruber* (13/262) and *Miniopterus inflatus* (1/249) species, were found infected with CoVs. We identified alphacoronaviruses in *H. gigas* and *H*. cf*. ruber* and betacoronaviruses in *H. gigas*. All *Alphacoronavirus* sequences grouped with *Human coronavirus 229E* (HCoV-229E). Ecological analyses revealed that CoV infection was significantly found in July and October in *H. gigas* and in October and November in *H*. cf *ruber*. The prevalence in the Faucon cave was significantly higher. Our findings suggest that insectivorous bats harbor potentially zoonotic CoVs; highlight a probable seasonality of the infection in cave-dwelling bats from the North-East of Gabon and pointed to an association between the disturbance of the bats’ habitat by human activities and CoV infection.

## Introduction

Coronaviruses (CoVs) belonging to the *Coronaviridae* family are viruses known to infect a wide range of animals and humans. In humans, CoVs are responsible for mild to severe respiratory illnesses such as the Severe Acute Respiratory Syndrome (SARS) and the Middle-East Respiratory Syndrome (MERS). The epidemic of SARS which started in 2002 in China, and spread on various other continents such as North America and Europe, reached a mortality rate of 9%^1^. The other severe epidemic illness (MERS) appeared more recently in the Middle East, and like the SARS, it also spread in other countries in Africa, America and Europe with case fatality rates of 35% (reviewed by de Wit *et al*.^2^). Currently the world is facing an ongoing pandemic caused by a new coronavirus (SARS-CoV-2) that emerged in China in December 2019 and caused disease (COVID-19) resulting in 118 326 confirmed cases and 4292 deaths as of March 11, 2020^3^. Since the emergence of these respiratory diseases, CoVs have been considered a real public health problem because of their ability to become epidemic and pandemic^4^. Even though human to human transmission of SARS-CoV and MERS-CoV occurs mainly through nosocomial transmission, some human cases recorded were occasionally the results of a zoonotic transmission^5^. This raised an interest in the identification of animal reservoirs. Indeed, it appears that almost each human CoV have zoonotic origins or otherwise have a close relative that circulate in wild animals (bats)^6–8^ and domestic animals (camels and cattle)^9,10^. Besides SARS-CoVs and MERS-CoVs, numerous other CoVs have been detected in bats in Africa, Asia, Europe and America and are classified into the genera *Alphacoronavirus* and *Betacoronavirus*^11,12^. For SARS-CoV-2, a recent study revealed that the whole genome sequence of SARS-CoV-2 was most closely related with a bat coronavirus detected in bats from Yunnan Province in China^13^. Consequently, bats were defined as reservoirs of ancestral coronaviruses which were transmitted to humans^14,15^. Other wildlife species appear to play an important role in the chain of transmission and the emergence of these viruses in humans, namely palm civets^5^ and wild rodents, in which infections by an alphacoronavirus and a betacoronavirus have been recently identified^16^.

Gabon, a country located in Central Africa, displays a large diversity of wildlife species. Aside from bats in which CoVs have been detected in the cave-dwelling species *Hipposideros* cf. *ruber*^17^, to our knowledge, there is no information on the carriage of other mammalian species. CoVs are prone to host switching^18^ and could be a current and future threat to public health.

The aim of this study was to explore the genetic diversity and the ecology of CoVs circulating among several wild mammals in Gabon in order to determine the potential reservoir species of these viruses and the risk for zoonotic emergence, and identify the factors driving CoVs infection in bats.

## Results

### Molecular identification of coronaviruses

A total of 1867 animal samples, including 1066 from bats of 5 different species, 494 from rodents of 16 different species, 33 from 4 species of primates, and 274 from 14 species of other wild animals, were screened for CoVs (Tables 1 and 2).

**Table 1:** Wild animal species analyzed by year and by locality.

Group	Province	City	Species	Year of sampling		
				2012	2013	2015
**Rodents**						
	Estuaire	Libreville	*Mus musculus*	—	23	—
			*Mus nannomys*	—	3	—
		Owendo	*Mus musculus*	—	54	—
			*Rattus rattus*	—	15	—
			undetermined	—	1	—
	Haut-Ogooue	Franceville	*Doemys ferrugiens*	6	—	—
			*Lemniscomys striatus*	9	—	—
			*Lophuromys ssp*	3	—	—
			*Mus nannomys*	4	—	—
			*Praomys sp*	12	—	—
			*Rattus rattus*	68	—	—
			*Stochomys longicaudatus*	3	—	—
		Leconi	*Cricetomys emini*	—	1	—
			*Lemniscomys striatus*	—	3	—
			*Lophuromys ssp*	—	1	—
			*Mus musculus*	—	8	—
			*Mus nannomys*	—	16	—
			*Praomys sp*	—	1	—
			*Rattus rattus*	—	38	—
	Ogooue-Ivindo	Makokou	*Cricetomys emini*	—	7	—
			*Grammomys poensis*	1	—	—
			*Hybomys univittatus*	4	—	—
			*Hylomyscus sp*	20	4	—
			*Lophuromys nudicaudus*	1	2	—
			*Malacomys longipes*	3	1	—
			*Mus musculus*	23	15	—
			*Mus nannomys setulosus*	7	—	—
			*Mus monticola*	1	—	—
			*Oenomys hypoxantus*	—	2	—
			*Praomys sp*.	40	15	—
			*Rattus rattus*	19	48	—
			*Stochomys longipes*	—	1	—
			undetermined	7	—	—
**Non-human primates**						
	Haut-Ogooue		*Cercopithecus pogonias*	—	1	—
			*Cercopithecus cephus*	—	4	—
			*Cercocebus torquatus*	—	2	—
	Ogooue-Ivindo		*Cercopithecus cephus*	—	1	—
			*Cercopithecus nictitans*	—	—	15
			*Colobus satanus*	—	—	24
	Ngounie		*Cercopithecus cephus*	—	1	—
			*Cercopithecus nictitans*	—	2	—
			*Cercocebus torquatus*	—	9	—
**Other wild animals**						
	Haut-Ogooue		*Eudorcas rififons*	—	5	—
			*Hystrix cristata*	—	4	—
			*Loxodont africana*	—	—	15
			*Manis javanica*	—	3	—
			*Strix aluco*	—	1	—
			*Syncerius caffer nanus*	—	—	49
	Ogooue-Ivindo		*Sylvicapra grimmia*	—	—	1
			*Cepahlophus monticola*	—	—	8
			*Cephalophus silvicultor*	—	—	3
			*Cephalophus dorsalis*	—	—	20
			*Civettictis civetta*	—	—	7
			*Eudorcas rififrons*	—	6	—
			*Genetta genetta*	—	—	1
			*Hypotragus equinus*	—	4	—
			*Hystrix cristata*	—	1	—
			*Loxodonta africana*	—	—	20
			*Manis javanica*	—	4	—
			*Potamochoerus porcus*	—	1	—
			*Syncerius caffer nanus*	—	—	15
			*Tragulus javanicus*	—	—	1
	Ogooue-Lolo		*Eudorcas rififons*	—	2	—
			*Hypotragus equinus*	—	2	—
	Ngounie		*Hystrix cristata*	—	2	—
			*Manis javanica*	—	1	—
			*Eudorcas rififrons*	—	6	—
			*Osteolaemus tetraspis*	—	1	—
			*Hypotragus equinus*	—	2	—
	Woleu-Ntem		*Manis javanica*	—	1	—
			*Eudorcas rififrons*	—	2	—
			*Hypotragus equinus*	—	1	—

**Table 2:** Species of bats analyzed by year and by locality.

Province	Cave	Species	Number of samples collected per year					Total	Number of bats infected with CoVs
			2009	2010	2011	2013	2014		
Haut-Ogooue	Djibilong								
		*Hipposideros cf. ruber*	—	—	—	5	—	5	0
		*Miniopterus inflatus*	—	—	—	69	—	69	0
		*Rousettus aegyptiacus*	—	—	—	8	—	8	0
Ogooue-Ivindo	Batouala								
		*Coleura afra*	10	—	—	—	—	10	0
		*Hipposideros* cf*. ruber*	58	—	—	—	—	58	2
		*Hipposideros gigas*	17	—	—	—	—	17	0
		*Miniopterus inflatus*	102	—	—	6	—	108	1
		*Rousettus aegyptiacus*	4	—	—	6	—	10	0
	Zadie								
		*Hipposideros* cf. *ruber*	46	16	25	1	—	88	0
		*Hipposideros gigas*	7	5	25	3	—	40	0
		*Rousettus aegyptiacus*	37	28	50	35	—	150	0
	Faucon								
		*Coleura afra*	—	34	50	18	—	102	0
		*Hipposideros* cf*. ruber*	57	13	25	16	—	111	11
		*Hipposideros gigas*	24	30	25	16	—	95	4
		*Miniopterus inflatus*	—	7	50	11	—	68	0
Ogooue-Lolo	Kessipoughou								
		*Hipposideros gigas*	—	—	—	—	4	4	0
		*Miniopterus inflatus*	—	—	—	—	4	4	0
		*Rousettus aegyptiacus*	—	—	—	—	119	119	0
			362	133	250	194	127	**1066**	**18**

**Figure 1:**
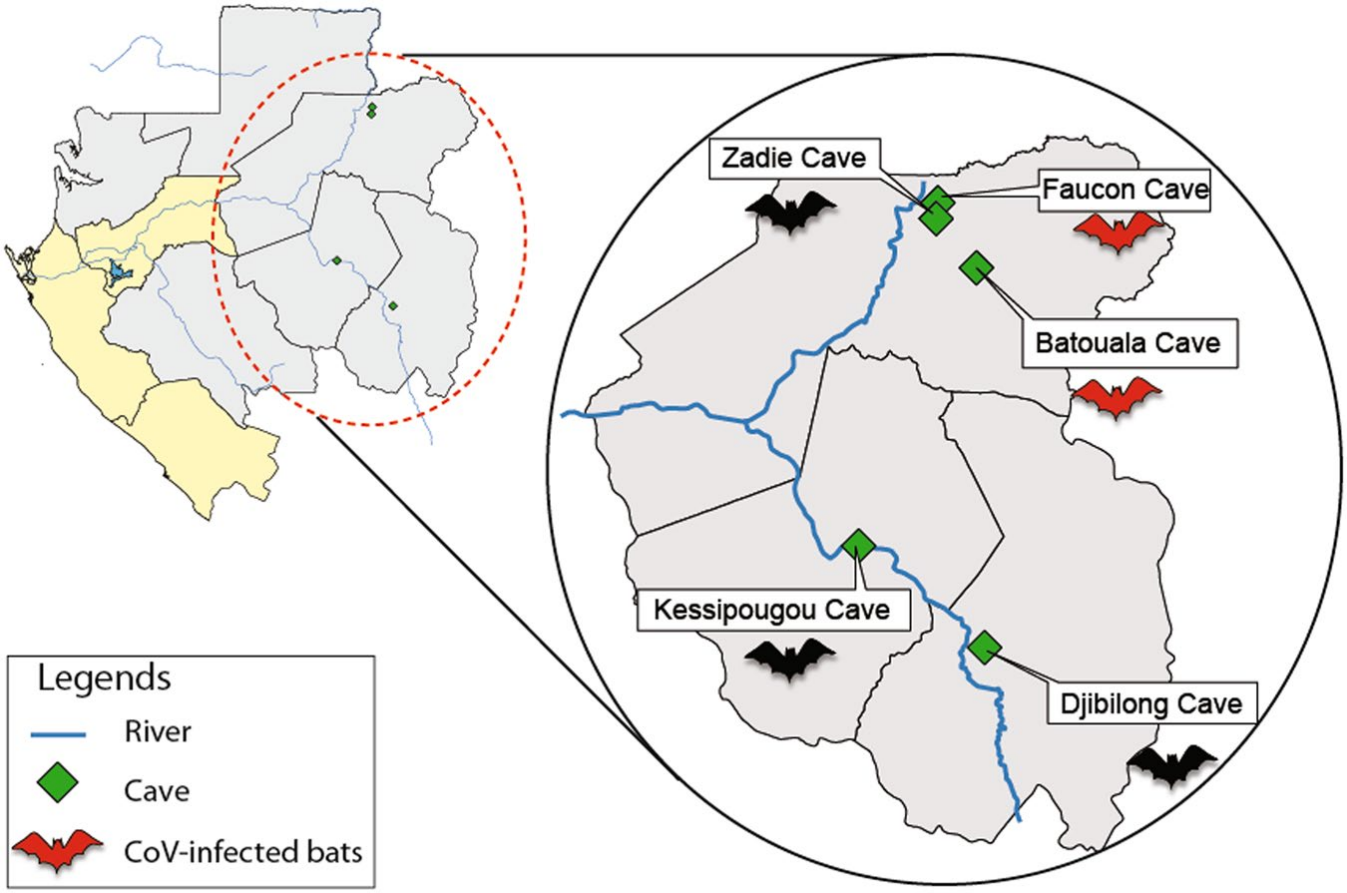
Location of animal sampling sites in Gabon.

Using nested RT-PCR, coronaviruses were detected in 18 intestine samples, giving an overall detection rate of 0.96% (18/1867). Positive samples originate from bats only, and the infection rate was 1.69% (18/1066). No CoVs were found in rodents, primates, or other terrestrial wild mammals. The 18 bat samples identified as CoV positive included 13 among the 262 *Hipposideros* cf*. ruber* (4.96%), 4 among the 156 *Hipposideros gigas* (2.56%), and 1 among the 249 *Miniopterus inflatus* (0.4%). All the positive bats came from the Ogooue-Ivindo province, in northeastern Gabon (Fig. 1), including 15 from the Faucon cave and 3 from the Batouala cave (Table 3). Besides CoVs detected in the Ogooué-Ivindo province and in the Faucon and Batouala caves, no other CoVs were detected in another province or in another cave. Bats positive for CoVs were captured in November and December 2009, October 2010 and July 2013. No individuals captured in 2011 and 2014 were found infected with CoVs. Both females and males were infected regardless of age.

**Table 3:** Characteristics of bats infected with Coronaviruses.

Bat ID	Genus	Species	Sex	Age	Location	Date of capture	Detected CoVs	Genbank accession number
09GB0274	*Hipposideros*	*cf. ruber*	F	SA	Faucon cave	November 2009	*Alphacoronavirus*	MG963194
09GB0296	*Hipposideros*	*cf. ruber*	F	SA	Faucon cave	November 2009	*Alphacoronavirus*	MG963195
09GB0323	*Hipposideros*	*cf. ruber*	M	SA	Faucon cave	November 2009	*Alphacoronavirus*	MG963192
09GB0328	*Hipposideros*	*cf. ruber*	F	SA	Faucon cave	November 2009	*Undetermined*	NA
09GB0329	*Hipposideros*	*cf. ruber*	M	SA	Faucon cave	November 2009	*Alphacoronavirus*	MG963193
09GB0376	*Hipposideros*	*cf. ruber*	M	SA	Faucon cave	November 2009	*Alphacoronavirus*	MG963196
09GB0379	*Hipposideros*	*cf. ruber*	M	SA	Faucon cave	November 2009	*Alphacoronavirus*	MG963197
09GB0380	*Hipposideros*	*cf. ruber*	F	SA	Faucon cave	November 2009	*Undetermined*	NA
09GB0383	*Hipposideros*	*cf. ruber*	F	SA	Faucon cave	November 2009	*Alphacoronavirus*	MG963191
09GB0761	*Hipposideros*	*cf. ruber*	M	A	Batouala cave	December 2009	*Alphacoronavirus*	MG963198
09GB0809	*Hipposideros*	*cf. ruber*	M	A	Batouala cave	December 2009	*Alphacoronavirus*	MG963189
09GB0812	*Miniopterus*	*inflatus*	M	A	Batouala cave	December 2009	*Undetermined*	NA
10GB0309	*Hipposideros*	*cf. ruber*	M	Juv	Faucon cave	October 2010	*Alphacoronavirus*	MG963199
10GB0318	*Hipposideros*	*cf. ruber*	F	Juv	Faucon cave	October 2010	*Alphacoronavirus*	MG963201
10GB0354	*Hipposideros*	*gigas*	M	A	Faucon cave	October 2010	*Alphacoronavirus*	MG963200
13GB0214	*Hipposideros*	*gigas*	M	A	Faucon cave	Jully 2013	*Betacoronavirus*	MG963186
13GB0215	*Hipposideros*	*gigas*	M	SA	Faucon cave	Jully 2013	*Betacoronavirus*	MG963187
13GB0273	*Hipposideros*	*gigas*	M	A	Faucon cave	Jully 2013	*Betacoronavirus*	MG963188

### Molecular characterization of identified CoVs

**Figure 2:**
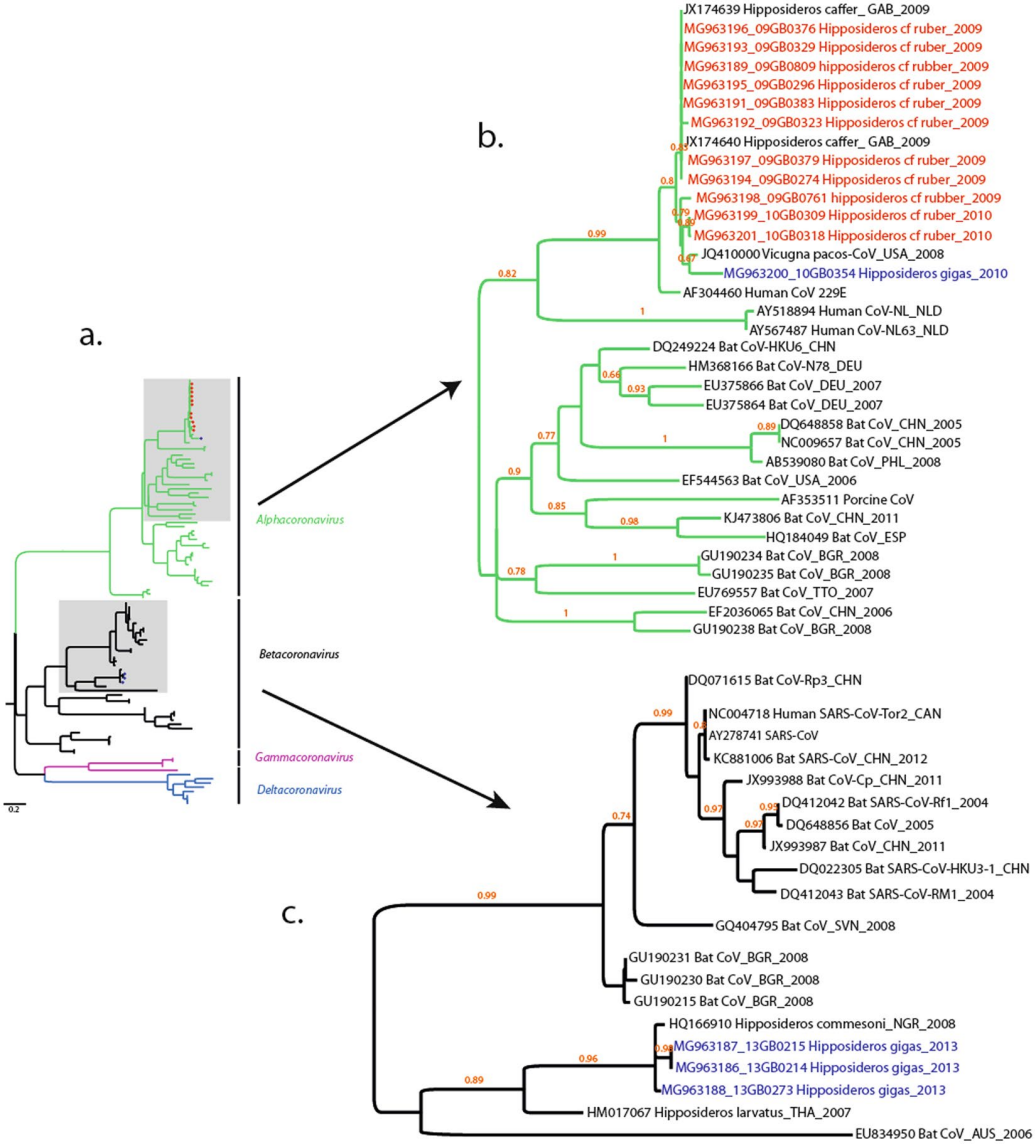
Phylogenetic relationship of coronaviruses based on a fragment of 495-bp of RdRP gene (**a**). Phylogenetic relationship between Alphacoronavirus (**b**), Betacoronavirus (**c**) and bat coronaviruses from this study are highlighted.

Fifteen bat nucleotide sequences were obtained and compared to those available in the public database using the algorithm “Blastn” of NCBI BLAST. The results indicated that all the nucleotide sequences from this study matched the deposited CoVs sequences. In order to determine the genetic relationships between the 18 bat CoVs from this study and previously described CoVs, phylogenetic analysis was performed based on 327-bp RdRp truncated sequences (Fig. 2a). The results showed that 12 out of 15 sequences belonged to the *Alphacoronavirus* (α-CoV) genus (Fig. 2b), whereas the remaining three belonged to the *Betacoronavirus* (β-CoV) genus (Fig. 2c). Among the 12 sequences of α-CoVs, 7 were obtained from individuals of *H. cf. ruber* caught in 2009 in the Faucon cave, 2 from other individuals of the same species but caught the same year in the Batouala cave; the other 3 sequences were obtained from 2 *H. cf. ruber* and 1 *H. gigas*, all caught in 2010 in the Faucon cave. The 3 sequences of β-CoVs were detected only in *H. gigas*.

The sequences of *Alphacoronavirus* were 91.9–100% identical with each other. The 7 sequences from 2009 from *H. cf. ruber* individuals of the Faucon cave showed 100% identity with each other. Regarding the 2 sequences of *Alphacoronavirus* from *H*. cf*. ruber* of the Batouala cave, the 09GB0761 (MG963198) sequence displayed either 96.5% or 96.8% with the 7 other sequences, while the 09GB0809 (MG963189) sequence showed 100% identity with the sequences from *H*. cf*. ruber* of the Faucon cave. Phylogenetic analysis supported this finding (Fig. 2b) and also showed that these 7 sequences clustered with 2 bat α-CoVs sequences (JX174639 and JX174640) from 2 *H*. cf. *ruber* from the Faucon cave caught in 2009. These two bat sequences showed 100% identity at nucleotide level with the 7 others. The 3 sequences of α-CoV (10GB0354, 10GB0309 and 10GB0318) from 2010 were 93.2–99.7% identical with each other. The 10GB0309 (MG963199) and 10GB0318 (MG963201) sequences, identical at 99.7%, from individuals of the same species (*H*. cf. *ruber*) caught in the Faucon cave, diverged respectively by 6.4% and 6.8% from the 10GB0354 (MG963200) sequence from an *H. gigas* captured in the same cave.

Furthermore, all α-CoV sequences grouped with the human coronavirus 229E (HCoV-229E) strain (AF304460). The nucleotide identity with HCoV-229E was 90.5–93.6%. Finally, the 10GB0354 sequence, from *H*. cf. *ruber* caught in the Faucon cave, showed 93.8% nucleotide identity with a CoV strain named Alpaca CoV (JQ410000), detected in *Vicugna pacos* commonly known as alpaca (family Camelidae).

The 3 sequences of β-CoV, 13GB0214 (MG963186), 13GB0215 (MG963187) and 13GB0273 (MG963188) were obtained from 3 *H. gigas* caught in 2013 in the Faucon cave. The 13GB0214 and 13GB0215 sequences displayed 99.7% identity at nucleotide level. These two sequences shared a nucleotide identity of 96.5% and 96.9% respectively with the 13GB0273 strain. In addition, these 3 sequences formed a distinct cluster with a bat β-CoV named the Zaria bat coronavirus (ZCoV) (accession number HQ166910), detected in Nigeria in 2008 in a species of bat genetically close to *H. gigas*, *Hipposideros commersoni*. The nucleotide identities between ZCoV and 13GB0214, 13GB0215 and 13GB0273 were 96.8%, 96.5% and 97.8%, respectively.

Bats of the species *H. cf. ruber* and *H. gigas* positive for alphacoronaviruses shared the same caves with the species *Coleura afra* and *Rousettus aegyptiacus*, which were negative for alphacoronaviruses. Moreover, the three *H. gigas* bats from the Faucon cave caught in 2013 (Table 3), shared the same cave with bats of the species *C. afra* and *M. inflatus* in which no *Betacoronavirus* was detected. Only the *H. gigas* species was infected with a *Betacoronavirus*.

**Figure 3:**
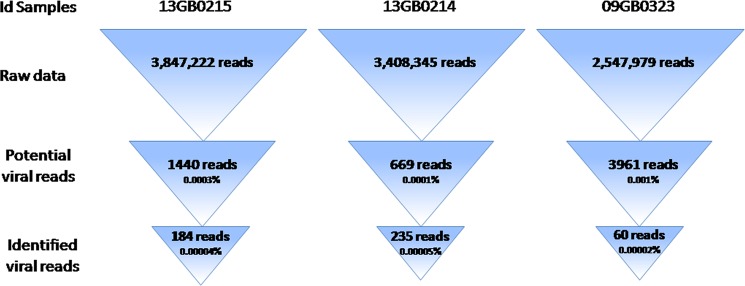
Identification of viral sequences from raw data generated by high-throughput sequencing. From the raw data, BLAST was performed against viral sequences from RefSeq to identify potential viruses. Then to discard false positive calls, reads having aligned with viral references were again aligned with NCBI Nucleotide database (nr).

High throughput sequencing yielded about 9.9 million reads (Fig. 3). Quality filtering discarded 86 955 (0.87%) sequences. No reads related to coronavirus could be detected with EDGE’s taxonomy classification module, but BLAST alignments (98–100% and 90–95%, for respectively query coverage and identity percentage) revealed 2 small coronavirus contigs (210 bp and 271 bp) from one sample. Those contigs have been determined as being parts of replicase polyprotein 1ab.

### Factors driving CoV positivity in bats

**Figure 4:**
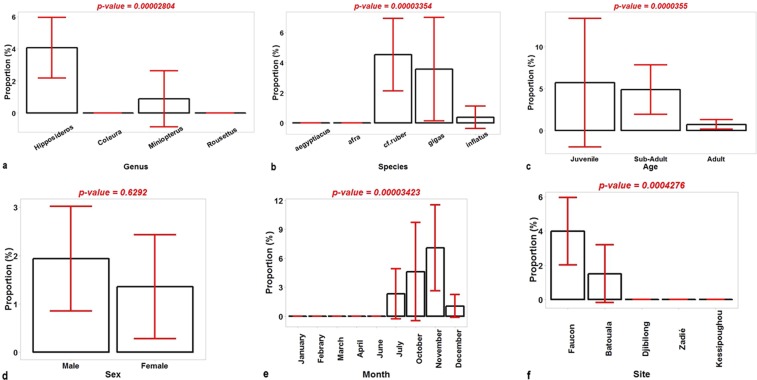
Comparison of the proportions of infected bats by genus (a), species (**b**), age (**c)**, sex (**d**), month (**e**) and site (**f**). The error bars represent the confidence interval.

**Figure 5:**
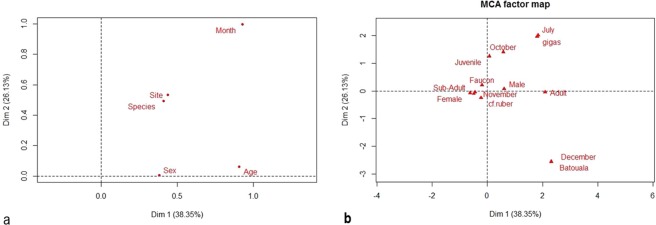
Multiple correspondence analysis on infected bats. Projection of the variables (**a**); projection of individuals (**b**).

The comparison of the proportions of infected bats by category for each variable showed that the differences observed between bat genera (Fig. 4a) were statistically significant (*p*-value = 0.00002804), as well as the variations within species (*p*-value = 0.00003354) (Fig. 4b), ages (*p*-value = 0.0000355) (Fig. 4c), months of capture (*p*-value = 0.00003423) (Fig. 4e) and sites (*p*-value = 0.0004276) (Fig. 3f). There was no significant difference between the 2 sexes (Fig. 4d). Furthermore, the MCA on all the bats revealed that the most important variables were the month of capture and the site followed by the species. In infected bats, the most important variables were also the month of capture, followed by the site and species (Fig. 5a). Figure 5b and Table 4 show the association between the categories of the variables and the characteristics of the different groups, respectively. According to these results, it appears that CoV infection in *H. gigas* is correlated with the months of July and October. In addition, infection with CoVs in the *H*. cf*. ruber* species can be associated with the months of November but also October. It appears that CoV infection is particularly associated with the Faucon cave.

**Table 4:** Characteristics of individuals in groups.

Groups	Categories					
	Genus	Species	Sex	Age	Site	Month
Group 1	*Hipposideros*	*caffer*	F	SA	Faucon cave	November
Group 2	*Hipposideros*	*caffer*	F/M	J	Faucon cave	October
Group 3	*Hipposideros*	*gigas*	M	A/SA	Faucon cave	July/October
Group 4	*Hipposideros/Miniopterus*	*caffe/inflatus*	M	A	Batouala cave	December

## Discussion

We studied 1867 animals from 39 species including rodents, bats, primates and other wildlife species, from 6 provinces (Estuaire, Haut-Ogooué, Ogooué-Ivindo, Ogooué-Lolo, Ngounié and Woleu-Ntem) in Gabon. This study identified coronaviruses (CoVs) in 18 bats belonging to the species *H. cf. ruber*, *H. gigas* and *M. inflatus* from two caves located in the Ogooue-Ivindo province located northeast of Gabon. The nucleotides sequences obtained showed that these CoVs belonged to the *Alphacoronavirus* and *Betacoronavirus* genera. In a previous study, the identification of CoVs was already reported in *H*. cf*. ruber* in Gabon^17^. Taken together, these results indicated that genetically distinct coronaviruses co-circulate among bats in the Ogooue-Ivindo province. These findings supported that bats carry a great genetic diversity of CoVs and Woo *et al*.^19^ suggested that bats were ideal hosts for both alphacoronaviruses and betacoronaviruses and could play an important role in the ecology and evolution of coronaviruses. Although no coronavirus was identified in other animal species, alpha- and betacoronaviruses were already found naturally in various wild mammals such as civets^5^, buffalos^20^ and rodents^21–23^. In their studies in rodents from China, Lau *et al*.^21^ and Wang *et al*.^22^ analyzed 725 and 1465 rodents, respectively, whereas we analyzed only 494 rodents. The lack of detection of CoVs in our study could be linked to the small sample size, unlike the aforementioned studies. However, in their study, Ge *et al*.^23^ found 23 infected rodents out of the 177 studied. Unfortunately the rodents in our study come mainly from urban localities, rodent captures should also be considered in rural forest areas around caves inhabited by bats. Moreover, experimental infections have been described in non-human primates^24,25^ but no natural infection of CoV in NHP have yet been reported. For other wild animals, the small size of samples investigated by species could explain the lack of CoVs found in this study.

Alphacoronaviruses and betacoronaviruses were detected only in *H*. cf. *ruber* and *H. gigas*, 2 among the 5 species tested. These results from the Ogooue-Ivindo province suggest that these 2 species could be both natural reservoirs of and specific for the alpha- and the betacoronavirus, respectively. A close co-evolutionary association could be suspected between CoVs, particularly betacoronaviruses, and bats of this family^26^. Interestingly, in our study, we detected a specific *Hipposideridae Betacoronavirus* clustering with a *Betacoronavirus* previously detected in Thaïland in 2007^26^. It was suggested that phylogenetic relationships between betacoronaviruses are mainly driven by the host phylogeny^26^. However, in a study conducted in Africa, Quan *et al*.^27^ suggested that there was no strict association between bat species and betacoronaviruses. Besides this study, previous studies on CoV host species in Asia have suggested that there was no strict association between bat species and alphacoronaviruses^28,29^. Indeed, many studies have described coronaviruses in a wide range of bat species^30,31^. Nevertheless, despite the report of alphacoronaviruses in numerous species of Miniopteridae and Vespertillionidae, Ar Gouilh *et al*.^32^ suggested a strict association between *Alphacoronavirus* EPI6 and Rhinolophidae, a familly usually associated with betacoronaviruses.

No CoVs were detected in chiropteran species sharing the same caves with the positive species (*H*. cf*. ruber* and *H. gigas*), suggesting a specificity of CoVs for these species or an absence of contact or interaction between some species. It was observed, for example, in the Faucon cave, that colonies of *H*. cf*. ruber* and *H. gigas* were found separately, in specific areas of the cave, the same with the other species (*M. inflatus* and *C. afra*), except for *R. aegyptiacus* which was not present in this cave. This spatial segregation could limit direct contact between species and represent a behavioral barrier to interspecific transmission, as previously described for lentiviruses and felids^33,34^ and more recently for bats and coronaviruses^32^.

A strain of *Alphacoronavirus* was identified in 7 *H*. cf. *ruber* nesting in the same cave (Faucon cave) and caught the same year (2009). Likewise, 2 other *H. cf. ruber* from the Batouala cave but caught in 2010 were infected by the same alphacoronavirus infecting the 7 *H*. cf. *ruber* from the Faucon cave. Corman *et al*.^14^ had already identified a closely related strain of *Alphacoronavirus* in 2 *H*. cf. *ruber* from the Faucon cave in 2009. Altogether, these findings first suggest the transmission of CoVs between individuals of the same species through close contacts probably increased by social and roosting behavior, large colony size and reduced space in these caves^26^; this strain of *Alphacoronavirus* could be enzootic in *H*. cf. *ruber* in this area of Gabon and roosts harboring a large number of bats nesting together promotes viral diffusion in the colony^29,35^, and second, local movements of bats individuals in the region imputable to seasonal/reproductive or opportunistic roosting behaviors, as the two caves being only 67 km apart. The migratory behavior of some bats provides an opportunity for pathogens to spread over long distances, as reported for flying foxes of the species *Eidolon helvum* able to migrate more than 1000 km^36^.

No individuals caught in 2011 and 2014 were found infected with CoVs. In 2011, only the guano of *C. afra*, *H*. cf. *ruber*, *H. gigas*, *M. inflatus* and *R. aegyptiacus* species was collected, unlike other years where organs were taken from bats. In this cave, the *R. aegyptiacus* colony lives in areas separated from other species including *H*. cf. *ruber* and *H. gigas*. The lack of detection in 2011 may be due to the poor quality of the samples having undergone several thawing. In 2014, only the *R. aegyptiacus* species was sampled in the Kessipoughou cave. In this cave, the *R. aegyptiacus* populations live in areas separated from other species including *H*. cf. *ruber* and *H. gigas*. However, Tong *et al*.^37^ and Lau *et al*.^30^ detected alphacoronaviruses in *Rousettus* bats in Kenya and China.

*Alphacoronavirus* strains detected in *H. cf. ruber* from the Faucon cave displayed a variable nucleotide identity of 91 to 93% with human coronavirus 229E. This virus causes a respiratory disease and is the cause of seasonal epidemics in humans^38^. In Ghana, Pfefferle *et al*.^6^ identified a strain of *Alphacoronavirus* in *H*. cf*. ruber* closely related to the human coronavirus 229E. Our finding argues that close relatives of the human coronavirus 229E exist in African bats^14^. The phylogenetic proximity of the CoV strains identified in this study with the human CoV-229E strain suggests a risk of emergence of this virus in humans in Africa. Furthermore, the 10GB0354 alphacoronavirus strain (MG963188), from *H. gigas* bats caught in 2010 in the Faucon cave, was phylogenetically close (93.8% nucleotide identity) to the Alpaca CoV strain detected in the *Vicugna pacos* species, commonly known as alpaca (JQ410000). Maganga *et al*.^17^ and Corman *et al*.^14^ already described two Gabonese CoV strains from hipposiderid bats caught in 2009 in the Faucon cave closely related to Alpaca CoV. Indeed, this coronavirus was described in 2007 during the epidemic of severe respiratory disorders associated with abortions in alpacas. It could be the direct progeny of its common ancestor with HCoV-229E, sharing with the latter a nucleotide identity of 92.2%^39,40^, on 440 bases of the nsp12 (polymerase). The phylogenetic link between these 2 CoVs could originate from an inter-species transmission, as a result of the opportunistic evolution of their common ancestor. As a result of genetic mutations and recombinations, the Alpaca CoV would have passed from bats to alpaca and *vice versa*. Phylogenetic analysis also revealed that two betacoronaviruses from 2 *H. gigas*, caught in 2013 in the Faucon cave, were phylogenetically close to a bat betacoronavirus named the Zaria bat coronavirus (ZCoV), detected in Nigeria in 2008 in a species of chiroptera genetically close to *H. gigas*, *H. commersoni*^27^, suggesting that phylogenetic proximity between host species may promote inter-species transmission^41^.

BLAST succeded in finding 2 contigs linked to coronavirus while no reads from this species could be detected by all EDGE’s taxonomy tools. To identify coronavirus reads having being assembled into those 2 contigs, further analyses using BLAST against NCBI RefSeq virus database were carried, allowing for the identification of 7 coronavirus reads. Since BLAST proved to be more sensitive, the same was done for the 2 other samples in order to verify that no coronavirus reads could have been missed. No more reads related to coronavirus were found. Moreover, this analysis overall showed that viral reads were extremely under-represented. One could argue that not finding the targeted virus might be caused by low DNA concentration in sequencing libraries. However, even in the sample where the highest concentration has been recorded, no coronavirus reads were found. Furthermore despite having achieved a satisfying quality sequencing, the biased amplification (majority species will be over-represented) and the low viral load explain why coronavirus was identified with such weak coverage.

Knowledge on the diversity of coronaviruses in bats and the factors promoting this diversity is important to prevent the risks of emergence in humans. Based on the analysis of data on life history traits of bats collected in the field, our findings suggest that the diversity of bat coronaviruses was associated with the seasonality and the site of capture, as well as the species of bats. It appeared that CoV infection in *H. gigas* correlated with the months of July and October, while CoV infection in *H*. cf. *ruber* was associated with the months of October and November.

In tropical ecosystems, some bat species seem to breed year-round, whereas others have annual or biannual birth peaks^42^. In Gabon, the parturitions in *H*. cf. *ruber* occur synchronously but some colonies give birth in March and others in October. In *H. gigas* from the Republic of Congo, a neighboring country, the young are born at the beginning of the rainy season. In Gabon, the months of October and November correspond to the beginning of the rainy season. A study demonstrated a high prevalence of CoVs in Chinese bats during the beginning of the rainy season^43^. The same finding was reported for the shedding of astroviruses in insectivorous bats in Asia (Borneo)^42^. Moreover, these months also correspond to the parturition season for *H. gigas* and *H*. cf*. ruber*. Indeed, lactating females of *H. cf. ruber* and juveniles of the same species were observed in October in northeastern Gabon. Regarding *H. gigas*, these observations are more difficult, Brosset and Saint Girons^44^ reported that the seasonal events of mating and parturition in *H. gigas* were confined in large caves where these large bats are entirely dependent to raise their young. According to Plowright *et al*.^45^, reproductive stress was an important driver of Hendra virus antibody prevalence. The prevalence of antibodies against the Hendra virus was higher in pregnant and lactating female of *Pteropus scapulatus* contrary to non-reproductive females. Different studies showed that parturition and lactation were important risk factors for CoVs shedding in insectivorous bats^46,47^. These two physiological states are energetically costly and thus lead to a depression of the immune system.

Moreover, our findings suggested that CoV infection was particularly associated with the Faucon cave. Of all the caves studied, the Faucon cave was the largest, with many cavities sheltering a diversity of bat species living in sympatry. This cave is regularly visited by villagers to hunt bats for human consumption. This hunting pressure could cause habitat disturbance and stress to these animals. Although Seltmann *et al*.^42^ found no association between habitat disturbance and the detection rate of CoVs in insectivorous bats, numerous studies in bats reported that pathogen prevalence was associated with habitat disturbance^48^. Many authors argued that habitat disturbance in some bats species may cause chronic stress and consequently immunosuppression^42,49–51^, increasing the susceptibility of animals to acquire and shed viruses.

In summary, the results of our study highlight a genetic diversity of CoVs in insectivorous bats in northeastern Gabon and show that the beginning of the rainy season and mainly the parturition season constitute a period of risk for CoV infection of insectivorous bats *H*. cf. *ruber* and *H. gigas*. These results also suggest an association between the disturbance of the bats’ habitat by human activities and the infection of these animals with these viruses. This study highlights a probable seasonality of the infection of insectivorous cave bats of the north-east of Gabon by CoVs. To conclude, a longitudinal monitoring should be carried out at different months of the year in order to determine the reproduction periods of the different bat species and therefore the periods favorable to viral shedding. The effect of the habitat disturbance on viral shedding should also be studied further. Bats harbor a large number of potentially zoonotic CoVs and pose a threat to public health for populations that are in contact with reservoir species of these viruses. The knowledge of CoVs ecology in bats is essential to prevent emergence in human populations.

## Methods

### Ethical approval

All experiments were performed in accordance with relevant guidelines and regulations. Experimental protocol was approved by the National Center of Scientific and Technologic Research (CENAREST) (permission number AR0031/09) for the use of non-human primate fecal samples. Fecal sample collection from non-human primates (NHPs) in protected areas was approved by the National Parcs Agency (ANPN). Bat and rodent captures and sampling were conducted with the permission of the Wildlife and Hunting Department of the Gabonese Ministry of Water and Forestry (N°003/MEFE-PA/SG/DGEF/DCF, N°0021/MEFE-PA/SG/DGEF/DCF and N°0031/MEFDD/SG/DGEF/DFC). All the capture events, animal handling, euthanasia and transfer of samples across the country were performed in accordance with the guidelines of the American Society of Mammalogists (http://www.mammalsociety.org/committees/animal-care-and-use). Safe and humane euthanasia was achieved through the use of halothane prior to autopsy. We performed the autopsies under a microbiological safety station equipped with personal protective equipment, mask and visor.

### Study area and sample collection

We screened several samples from bushmeat, bats, rodents and NHPs collected from 2009 to 2011 and from 2013 to 2015 in six provinces of Gabon (Fig. 1).

#### Bushmeat sampling

Bushmeat was collected from officers of the provincial direction of the Gabon Ministry of Water Affairs and Forestry after seizures of illegal hunting, as well as from salesmen during the hunting season. The sampling was performed in 2013 in four provinces of Gabon (Haut-Ogooué, Ngounié, Ogooué-Ivindo and Woleu-Ntem). Species identification was performed as previously described^52^. Samples, including whole blood, liver, spleen and gut, were then kept in liquid nitrogen until their delivery at the CIRMF (*Centre International de Recherches Médicales de Franceville*) and stored at −80 °C for further laboratory investigations.

#### Bat and rodent sampling

Bat capture took place in three provinces of Gabon, namely Ogooué-Ivindo, Ogooué-Lolo and Haut-Ogooué in 2009, 2010, 2011, 2013 and 2014. In Ogooué-Ivindo, capture was performed in three caves, the Faucon (1°07 N 13°20 E), Zadie (0°98 N 13°19 E) and Batouala (0°82 N 13°45 E) caves, located in the heart of the Belinga mountains. The distances between the three different caves located in Belinga were as follows: 33.7 km between the Batouala and Zadie caves, 11.7 km between the Zadie and Faucon caves and 37.7 km between the Batouala and Faucon caves^53^.

In the Ogooué-Lolo province, bats were caught in the Kessipoughou cave (0°86 S 12°77 E); and in the Haut-Ogooué province, bats were captured in the Djibilong cave (1°36 S 13°46 E), located north of Franceville. For bats, sex, stage of maturity or age (adult, subadult, juvenile) and capture season were recorded. Rodents were sampled in 2012 and 2013, using Tomahawk and Sherman traps as previously described^54^. The traps were set in houses and their vicinities in three provinces of Gabon: Estuaire (Libreville and Owendo), Ogooué-Ivindo (Makokou) and Haut-Ogooué (Franceville and Leconi). After autopsy, organs were collected in bats and rodents, frozen and then stored at −80 °C until analysis. Bat and rodent species were identified by trained field biologists.

#### Feces collection

Fecal samples were collected from NHPs, bats and wild animals. Fresh bat droppings were collected on plastic film arranged below roost sites in the caves^55^. Additionally, fecal pellets were directly collected in bags in which the bats were put individually after capture. Fecal samples from NHPs were collected in the field in the Ogooué-Ivindo province (Lope National Park) in 2015. Feces from other wild animals were sampled in 2015 in the Haut-Ogooué (Lekedi Park in Bakoumba) and Ogooué-Ivindo (Lope National Park and Mopia) provinces. Fresh fecal samples were preserved in approximately 10 ml of RNA*later* (Ambion) or frozen and transferred to the CIRMF laboratory, where they were stored at −80 °C until analysis.

### Specimen preparation and RNA extraction

Total RNAs were extracted from the intestines of bats and rodents coming from bushmeat, and from the feces of bats, PNHs and other wild animals. Around 100 mg of intestine was pooled according to the species and crushed in 600 µl of cold phosphate buffer saline (PBS) (Biological Diagnostic Supplies Ltd) in a ball-mill tissue grinder (Geno/Grinder 2000, Spex Centripep). Total RNA was extracted from 100 µl of homogenate supernatant using a Biorobot EZ1 and the EZ1 RNA tissue mini kit (Qiagen) according to the manufacturer’s guidelines in a BSL3. For feces preserved in RNA*later* solution, RNA*later* was taken out before feces was suspended in PBS and centrifuged at 1500 rpm for 5 min. RNAs were then extracted from 400 µl of supernatant, using the EZ1 RNA Viral Mini kit (Qiagen) according to the manufacturer’s recommendations.

The RNA was then tested for the detection of CoVs using a nested reverse transcription-polymerase chain reaction (nRT-PCR)^56^ using generic primers targeting a 494 bp fragment of a conserved region of the RNA-dependant RNA polymerase (RdRp) gene. The first-round PCR was carried out using the Qiagen One-step RT PCR kit (Qiagen). Amplification involved 30 min at 50 °C; 15 sec at 95 °C, followed by a touchdown element of 10 cycles of 20 sec at 94 °C, 30 sec starting at 60 °C with a decrease of 0.5 °C per cycle, and 40 sec at 72 °C; and 40 cycles of 20 sec at 95 °C, 30 sec at 54 °C, 40 sec at 72 °C, with a final elongation step of 5 min at 72 °C. The second-round PCR was performed using the *Platinum Taq* DNA polymerase kit (Life Technologies). Amplification involved 3 min at 94 °C followed by 40 cycles of 20 sec at 94°, 30 sec at 60 °C, 40 sec at 72 °C, with a final elongation step of 1 min at 72 °C. Individual samples from pools which gave positive results were tested separately by the same nRT-PCR.

### Hight-throughput sequencing

The hight-throughput sequencing was performed using a MiSeq machine. The DNA library was prepared from 3 samples (13GB0214, 13GB0215 and 09GB0323) using the NEBNext Ultra RNA Library Prep kit for Illumina (New England BioLabs, UK) following the manufacturer’s recommandations. Briefly, after RNA quantification and fragmentation, cDNAs were generated with SuperScript III reverse transciptase. Agencourt AMpure XP beads (Beckmann Coulter, The Netherlands) was used for size selection purification and resulted in a total library size 300–500 bp. Library quality was evaluated on an Agilent BioAnalyser 2100 (Agilent Technologies, USA). Sequencing was performed on Illumina’s MiSeq (V2 reagents) to obtain 2 ×150 bp paired-end reads. Bioinformatic analyses were conducted with EDGE v2.3.1 pipeline^57^. EDGE’s quality module (FastQC v2.08 tool) filtered sequencing reads based on a 25 PHRED score threshold, no ambiguous bases and a 50 bp minimum size. Then taxonomy classification was conducted on both trimmed reads and contigs after assembly (SPAdes v3.13.0). The EDGE’s taxonomy module allows for a broad exploration using minimap2 v2.16, Kraken2 v2.0.7, Metaphlan v2.7.7, BWA v0.7.12 and GOTTCHA2 v2.1.5. Unclassified contigs were aligned with BLAST^58^ and NCBI RefSeq virus database (September 16, 2019 release).

### Phylogenetic analyses

The partial RdRp sequences obtained were cleaned and the contigs assembled using ChromasPro 1.5. The nucleotide sequences were compared to those available in GenBank using the “Blastn” algorithm of NCBI BLAST. Multiple sequence alignments were performed using the ClustalW algorithm implemented in the MEGA 7 software^59^. Phylogenetic analyses were performed by maximum likelihood algorithm using 1,000 bootstraps replications with PhyML^60^ and also under a Bayesian statistical framework implemented in BEAST (version 1.8.3)^61^ using the general time reversible model of substitution, with a gamma distribution and a proportion of invariant sites (GTR + I + G).

### Nucleotide sequence accession numbers

All the sequences obtained in this study have been deposited in Genbank under the accession numbers MG963186-MG963189 and MG963191-MG963201.

### Statistical analysis

Bat data were analyzed for factors associated with CoV positivity. The methodology is based on the combined use of multiple correspondence analysis and hierarchical clustering. First, multiple correspondence analysis (MCA) was done on all the individuals, and then only on the positive bats to better identify these factors. For each variable included in the analyses (age, sex, bat genus and species, month of capture, site) (Table 5), the proportions of the infected animals were compared using the Chi square test. A *p* value of <0.05 was considered for significant results. A characterization of the infected bats was made through a hierarchical clustering by the k-means method. The data were analyzed using R version 3.4.3.

**Table 5:** Description of risk factors for infection of cave-dwelling bats with CoVs.

Variables	Description	No. of animals
**Species**		
	*Coleura afra*	112
	*Hipposideros cf. ruber*	262
	*Hipposideros gigas*	156
	*Miniopterus inflatus*	249
	*Rousettus aegyptiacus*	287
**Sex**		
	Male	620
	Female	443
	NA	3
**Sites/Caves**		
	Batouala	203
	Djibilong	82
	Faucon	376
	Kessipoughou	127
	Zadie	278
**Months**		
	January	65
	February	127
	March	93
	April	129
	June	101
	July	112
	October	77
	November	74
	December	288
**Age**		
	Adult	823
	Subadult	205
	Juvenile	35
	NA	3
